# Telephone-assisted CPR: A literature review

**DOI:** 10.1007/s10049-016-0210-5

**Published:** 2016-08-05

**Authors:** M. Maier, M. Luger, M. Baubin

**Affiliations:** Univ.-Klinik für Anästhesie und Intensivmedizin Tirol Kliniken, Medizinische Universität Innsbruck, Anichstraße 35, 6020 Innsbruck, Austria

**Keywords:** Cardiopulmonary resuscitation, Cardiac arrest, Cardiac massage, Bystander resuscitation, Emergency medical services, Kardiopulmonale Reanimation, Herzstillstand, Herzdruckmassage, Laienreanimation, Notfallmedizin

## Abstract

**Introduction:**

Despite numerous efforts, out-of-hospital cardiac arrest (OHCA) survival has not significantly increased in recent decades. The first telephone-assisted cardiopulmonary resuscitation (T-CPR) studies were published in the 1980s, but only in the last decade has T‑CPR been implemented in dispatch centers. T‑CPR is still not available in all dispatch centers and no national or international T‑CPR recommendations are available.

**Methods:**

Studies from PubMed were identified and evaluated. Preliminary information from the European Dispatch Center Survey (EDiCeS) is also included.

**Results:**

In all, 42 studies were included. T‑CPR is implemented in 87.6 % of those dispatch centers which have joined the not-yet published EDiCeS. According to German Resuscitation Registry data, about 10 % of OHCA patients received T‑CPR in 2014. Agonal breathing is the leading cause for nonrecognition of OHCA by the dispatcher. Sensitivity of OHCA recognition by the dispatcher is about 75 %, whereby 8–45 % of these patients were not in cardiac arrest. The time interval from call to first compression is 140–328 s. Instructing rescue breathing by telephone is time consuming, leads to extensive hands-off times, and often to ineffective ventilation; therefore, rescue breathing is not indicated in adults with primary cardiac arrest. Studies showed improved survival with standardized T‑CPR implementation.

**Conclusion:**

T-CPR is established in many dispatch centers. However, emergency call interrogation and T‑CPR vary between dispatch centers and are often performed without evaluation. International recommendations with standardized quality control are necessary and may lead to improved survival.

## Zusammenfassung

**Einleitung:**

Trotz zahlreicher Bemühungen zeigte sich in den letzten Jahrzehnten kein signifikanter Anstieg der Überlebensrate nach außerklinischem Herz-Kreislauf-Stillstand (HKS). Erste Studien zur Telefonreanimation (T-CPR) wurden bereits in den 1980er Jahren durchgeführt, die Einführung in die tägliche Praxis erfolgte jedoch größtenteils im letzten Jahrzehnt. Allerdings ist die T‑CPR bis heute nicht flächendeckend in allen Leitstellen verfügbar, und es existieren weder nationale noch internationale Empfehlungen.

**Methoden:**

Studien aus PubMed wurden mit vordefinierten Suchbegriffen identifiziert und evaluiert. Des Weiteren sind erste Ergebnisse aus der „European Dispatch Center Survey“ (EdiCeS) in diesen Artikel eingeflossen.

**Ergebnisse:**

Insgesamt wurden 42 Studien zitiert. Aus der noch nicht publizierten EdiCeS-Studie wird T‑CPR in 88 % der teilnehmenden Leitstellen angewendet. Laut deutschem Reanimationsregister wurden im Jahr 2014 10 % der außerklinischen HKS telefonisch angeleitet. Die Sensitivität zur Erkennung eines HKS durch den Leitstellendisponenten liegt bei ca. 75 %. Dabei ist Schnappatmung die häufigste Ursache für das Nichterkennen eines HKS. Hingegen befinden sich 8–45 % der Patienten, bei welchen T-CPR angeleitet wurde, im HKS. Die Zeit vom Anruf bis zur ersten angeleiteten Thoraxkompression liegt bei 140–328 s. Die telefonische Anleitung zur Mund-zu-Mund-Beatmung ist zeitintensiv, führt zu verlängerten Hands-off-Zeiten und ist bei Laien häufig ineffektiv – daher ist sie bei Erwachsenen mit kardial bedingtem HKS nicht indiziert. Studien zeigen, dass eine standardisierte Anwendung der T‑CPR die Überlebensrate bei HKS verbessern kann.

**Schlussfolgerung:**

Die T‑CPR hat sich in der Mehrzahl der Leitstellen etabliert. Allerdings variieren das Abfrageschema und die angeleitete T‑CPR stark, und die Prozesse werden oft ohne Evaluierung und Qualitätsmanagement durchgeführt. Internationale Empfehlungen mit standardisiertem Qualitätsmanagement sind dringend erforderlich. Hierdurch kann die Überlebensrate verbessert werden.

## Introduction

In 1960, Kouwenhoven et al. [1]* described combined chest compression and rescue breathing as basic life support – cardiopulmonary resuscitation (BLS-CPR) for the first time. In 1974, the first recommendations for CPR [2]* were issued and since then international CPR guidelines have been published at regular intervals. Despite a huge number of studies, frequent guideline updates, and international networks, survival rates have essentially not increased in the last few decades [3–5]*.

Immediate bystander CPR on the scene leads to a significant increase of quantitative and qualitative outcome in sudden cardiac arrest patients with double to fourfold survival rates [5–7]*. However, training laypersons in CPR is time-consuming and has to be refreshed periodically; instructions on CPR via telephone through a dispatcher (T-CPR) were proven to be an effective and reasonable method to improve the rate of bystander-performed resuscitation [8–13]. In a 2008 literature review, telephone-assisted CPR was shown to have the greatest potential to increase layperson resuscitation [14].

The fact that T‑CPR is a powerful tool for resuscitation improvement was already postulated by Eisenberg and Carter in the 1980s [10, 15, 16]. However, T‑CPR was not anchored in the ERC guidelines [17]* before 2010 and it took multiple studies before T‑CPR obtained a key position in the 2015 ERC guidelines [6]. Nevertheless, data on T‑CPR prevalence are still very rare. Much must still be done for T‑CPR to become well standardized and for comprehensive implementation.

The aim of this article is to give an overview of the extensive T‑CPR topic.

## Methods

Studies from the online database PubMed from 1984–2016 were identified using the search criteria “cardiac arrest telephone”, “cardiac arrest dispatch”, and “dispatcher cpr”; also included in the evaluation were eight articles from PubMed about CPR in general which are marked in this article with an asterisk (*). In addition, preliminary information from the European Dispatch Center Survey (EDiCeS) abstract by M. Luger presented at 2015 ERC Resuscitation in Prague are included.

## Results

The search criterion “cardiac arrest telephone” resulted in 359, “cardiac arrest dispatch” in 220, and “dispatcher cpr” in 147 hits for a total of 726 hits. For a comprehensive overview, these publications were evaluated looking for data on T‑CPR incidence, criteria for detecting out-of-hospital cardiac arrest (OHCA) by emergency medical dispatchers (EMD), sensitivity and specificity, time delay caused by T‑CPR, T‑CPR protocols, EMD training, T‑CPR with vs. without ventilation and on T‑CPR outcome. Another 42 publications were considered for further analyses.

### T-CPR incidence

No literature result was found for T‑CPR incidence and little is known about T‑CPR implementation and its practice across Europe and the USA.

Thus, EDiCeS was initiated with the aim to record data from many European dispatch centers (DC) via an online survey. From the 20 involved European countries, 91 DCs and/or dispatch organizations participated in the study covering regions with a total population of 108.7 million, thus, representing 23.7 % of the involved countries’ population and 13.4 % of Europe’s 742.5 million total population. With 87.6 % of the 91 consulted DCs, the results indicate a high percentage of T‑CPR implementation in Europe.

From the on-scene perspective and according to the German Resuscitation Registry (GRR) reports from 2007–2014, the rate of layperson CPR increased with an important part of T‑CPR provided on the patient. According the 2016 publication from Bohn et al. [18] who analyzed data of the GRR, the rate of T‑CPR increased from nearly zero in 2007 to about 10 % in 2014. During the same period, the recorded bystander CPR rate increased from 19 to 31 %. Therefore, every third bystander CPR in Germany is now telephone assisted. However, compared to Sweden with a rate of 55 %, the bystander CPR rate in Germany is still low [19]*.

Dami et al. [20] published in a study from the Swiss Canton Waadt a T-CPR rate of 29 % in a well-established T‑CPR system. Included were 1254 patients with assumed primary cardiac OHCA and the following reasons for not performed T‑CPR were compiled:

- The dispatcher had the opportunity to assess cardiac arrest in 85 % (*n* = 1072). In the remaining 15 % (*n* = 182), the caller was not on site or the patient was conscious at the time of the call.
- In 71 % (*n* = 895), cardiac arrest was recognized by the dispatcher. The main reason for not recognizing OHCA was insufficient assessment of the patient’s breathing.
- In 54 % (*n* = 683), T‑CPR was proposed by the dispatcher. The reasons for not proposing T‑CPR by the dispatcher were evident death and bystander spontaneous CPR.
- In 34 % (*n* = 429), the bystander accepted T‑CPR but then the bystander declined T‑CPR because the bystander was unable to move the patient (*n* = 89), because of stress/emotion (*n* = 66), because the bystander believed the patient was dead (*n* = 45) or because the caller was not on the scene any more (*n* = 21).
- As described above, T‑CPR was performed in 29 % (*n* = 364).

The same author reported in 2010 that the bystander’s physical condition was responsible for 58 % and emotional distress for 35 % of cases in which CPR was not carried out, when medically appropriate reasons for not performing CPR were excluded. Medically appropriate reasons for not performing CPR were ambulance arrived before resuscitation could be started, bystander was remoted from scene, patient with terminal illness, bystander believed patient is alive or prolonged down time [21].

### Criteria for detecting OHCA by emergency medical dispatchers

According to the 2015 ERC Guidelines, OHCA has occurred if a patient is unconscious and not breathing normally [6]. For the layperson the recognition of “not normal” breathing is difficult because up to 45 % of the OHCA patients show “agonal breathing” at the time the emergency call is taking place [9, 22, 23]. The distinction between agonal breathing and normal breathing is difficult for the layperson at the scene. This is the main reason why T‑CPR is instructed in over 92 % in case of apnea, whereas in 23 % in case of “agonal breathing” [22]. In another study, agonal breathing is also claimed to be the main reason for the not correctly recognized cardiac arrest by EMDs [24].

### Sensitivity and specificity

According to the literature, sensitivity of dispatcher-detected OHCA is stated in about 75 % [9, 20, 24–30] with a high fluctuation ranging from 56 % [9] to 97 % [30]. According to Dami et al. [20], poor breathing assessment was the cause of more than half of the missed OHCA cases.

The percentage of non-indicated T‑CPR instructions in patients being not in cardiac arrest vary from 8–45 % [25, 29, 31–33]. The rate of 45 % (*n* = 762) of non-indicated T‑CPR instructions in the study from White et al. [31] seems to be high but fortunately only 41 % (*n* = 313) of these patients were not in cardiac arrest and received bystander chest compressions. Luckily, consequential injuries are very rare. Of these patients, 12 % later suffer from “discomfort”, while 2 % report skeletal injuries such as rip or clavicular fractures. Organ injuries or deaths have not been reported [31, 32].

### Time delay caused by T‑CPR

According to the reviewed literature, the time interval from emergency call to dispatchers OHCA recognition is 60–170 s and from call to first instructed compression 140–328 s [8, 20, 26, 27, 33, 34]. Instructions on chest compression combined with rescue ventilation take 1.4 min longer than chest-compression-only instructions alone [35].

### T-CPR protocols, EMD-training and -experience

Identification of OHCA, bystander instructed CPR, and even quality of T‑CPR can be considerably enhanced by using standardized interrogation protocols and quality assurance programs in the dispatch centers [28, 36–39]. In a study from London [36], there was a 200 % rise in the number of patients accurately identified as suffering from cardiac arrest after introduction of the Advanced Medical Priority Dispatch System.

Furthermore, the patient’s survival in case of OHCA correlates with the dispatcher’s experience and number of processed T‑CPRs. In a study from Finland [26], the hospital discharge rate was 22 % if the individual dispatcher executed less than four T‑CPRs per year, while with experience of four or more T‑CPRs per year the rate increased to over 38 % (*p* = 0.02).

### T-CPR with vs. without ventilation

Three large-scaled randomized controlled trials were set up to show a superiority of T-CPR with chest-compression-only [35, 40, 41]. All three studies showed a trend toward better outcome for T-CPR with chest-compression-only but in none of the three studies was the result clinically significant. A meta-analysis of these studies, performed by Huepfl et al. [42], found a significantly improved chance of survival with chest-compression-only T‑CPR (survival: 14 %) compared with standard T‑CPR (survival: 12 %) with a number needed to treat of 41 patients.

### T-CPR outcome

In various studies, T‑CPR was found to increase the rate of bystander CPR [8–13]. Bystander CPR is proven to increase OHCA survival by 2–4 times [5–7]. Besides the fact that large-scaled, prospective and randomized trials are lacking, it may be obvious—remembering the facts listed above—that T‑CPR has the potential to raise survival. In a retrospective analysis from South Korea, T‑CPR showed clearly survival benefits. After implementation of T‑CPR survival rates increased from 7.1 to 9.4 % (*p* = 0.001) and good neurological outcome nearly doubled [13].

In a Finnish study from Helsinki, signs for efficiency of T‑CPR were shown by Kuisma et al. [26]. In patients with ventricular fibrillation, the survival rate until hospital discharge was 43 % when CPR instructions were given and 32 % when not given. A Japanese study with 1780 children also found a significant increase of 1‑month survival rate with T‑CPR instructions (OR 1.46), but there was only a nonsignificant effect on the improvement in favorable neurologic outcome at 1 month [12].

## Discussion

According to the available literature, telephone-assisted CPR by experienced dispatchers in a structured dispatch center with defined T‑CPR algorithms has the potential to significantly increase outcome after OHCA regarding number and quality. T‑CPR is a modern and promising concept within the emergency process, but it is not implemented area-wide yet. According to recent study results, consistent implementation with regular quality control must be considered urgent, otherwise it could be judged as an organizational fault. T‑CPR implementation is simple and not associated with enormous material or personnel costs.

The interval from the first telephone ring in the dispatch center to the first advised chest compression often takes too long and varies substantially between studies and the used technique in several dispatch centers. Clearly defined, standardized, and validated interrogation protocols for recognition of cardiac arrest and defined guidance for chest compression measures will decrease the time interval until the first advised chest compression.

Beside other reasons—but mainly caused by the difficult recognition of agonal breathing in OHCA—about 25 % of OHCA situations are not identified by the dispatcher. In contrast, 8–45 % of T‑CPRs which are advised by the dispatcher are not indicated from the point of view of the later arriving EMS professionals. For whatever reason it is a fortunate circumstance that in these patients who are not in cardiac arrest T‑CPR is started about half as often as the instructions are given. Up to now, no sustained harm has happened to these patients. Specificity and sensitivity of the interrogation especially with regard to agonal breathing can be improved via a standardized procedure. Therefore again, it is obvious that a standardized T‑CPR procedure is mandatory.

The studies clearly show that mouth-to-mouth ventilation by untrained laypeople is ineffective and ventilation instructions via telephone are very time-consuming. Hence, in OHCA with assumed cardiac origin, chest-compression-only is the method of choice in the initial CPR phase. In children, special situations like drowning, and after about 8 min of apnea, artificial ventilation is also indicated and may be advised to bystanders who are willing to perform.

Some T‑CPR limitations must be mentioned. If a linguistic or communication problem between the bystander and the dispatcher exists or the bystander is not capable or willing to perform CPR measures, CPR instructions do not make any sense. If the bystander is not able to lift the patient (e. g., from the bed to the floor), if the room is very constricted (e. g., in the bathroom), or if the communication goes via landline (e. g., from another room) or if the bystander uses his mobile phone but is not experienced to activate the external loud speaker, T‑CPR success will be limited.

As no recommendations on the topic T‑CPR exist, indications, content, procedure, and quality control differ widely. Thus, the following are urgently needed and important to improve the chain of survival: preparation and publishing of universal key recommendations for T‑CPR, establishment of interprofessional cooperation and networks, and performing high quality studies to point out strengths and weaknesses of telephone-assisted CPR.

## References

[1] Kouwenhoven WB, Jude JR, Knickerbocker GG (1960). Closed-chest cardiac massage. JAMA.

[2] Carveth S (1974). Editorial: Standards for cardiopulmonary resuscitation and emergency cardiac care. JAMA.

[3] Engdahl J (2003). Time trends in long-term mortality after out-of-hospital cardiac arrest, 1980 to 1998, and predictors for death. Am Heart J.

[4] Rea TD (2003). Temporal trends in sudden cardiac arrest: A 25-year emergency medical services perspective. Circulation.

[5] Sasson C (2010). Predictors of survival from out-of-hospital cardiac arrest: A systematic review and meta-analysis. Circ Cardiovasc Qual Outcomes.

[6] Perkins GD (2015). Part 3: Adult basic life support and automated external defibrillation: 2015 International Consensus on Cardiopulmonary Resuscitation and Emergency Cardiovascular Care Science with Treatment Recommendations. Resuscitation.

[7] Hasselqvist-Ax I (2015). Early cardiopulmonary resuscitation in out-of-hospital cardiac arrest. N Engl J Med.

[8] Culley LL (1991). Dispatcher-assisted telephone CPR: Common delays and time standards for delivery. Ann Emerg Med.

[9] Vaillancourt C (2007). Evaluating the effectiveness of dispatch-assisted cardiopulmonary resuscitation instructions. Acad Emerg Med.

[10] Eisenberg MS (1985). Emergency CPR instruction via telephone. Am J Public Health.

[11] Tanaka Y (2012). The continuous quality improvement project for telephone-assisted instruction of cardiopulmonary resuscitation increased the incidence of bystander CPR and improved the outcomes of out-of-hospital cardiac arrests. Resuscitation.

[12] Akahane M (2012). Impact of telephone dispatcher assistance on the outcomes of pediatric out-of-hospital cardiac arrest. Crit Care Med.

[13] Song KJ (2014). Dispatcher-assisted bystander cardiopulmonary resuscitation in a metropolitan city: A before-after population-based study. Resuscitation.

[14] Vaillancourt C, Stiell IG, Wells GA (2008). Understanding and improving low bystander CPR rates: A systematic review of the literature. CJEM.

[15] Carter WB (1984). Development and implementation of emergency CPR instruction via telephone. Ann Emerg Med.

[16] Eisenberg MS (1986). Identification of cardiac arrest by emergency dispatchers. Am J Emerg Med.

[17] Koster RW (2010). European Resuscitation Council Guidelines for Resuscitation 2010 Section 2. Adult basic life support and use of automated external defibrillators. Resuscitation.

[18] Bohn A, Seewald S, Wnent J (2016). Resuscitation – Basic life support in adults and application of automatic external defibrillators. Anasthesiol Intensivmed Notfallmed Schmerzther.

[19] Stromsoe A (2010). Education in cardiopulmonary resuscitation in Sweden and its clinical consequences. Resuscitation.

[20] Dami F (2015). Time to identify cardiac arrest and provide dispatch-assisted cardio-pulmonary resuscitation in a criteria-based dispatch system. Resuscitation.

[21] Dami F (2010). Why bystanders decline telephone cardiac resuscitation advice. Acad Emerg Med.

[22] Bohm K (2007). Dispatcher-assisted telephone-guided cardiopulmonary resuscitation: an underused lifesaving system. Eur J Emerg Med.

[23] Vaillancourt C (2011). In out-of-hospital cardiac arrest patients, does the description of any specific symptoms to the emergency medical dispatcher improve the accuracy of the diagnosis of cardiac arrest: A systematic review of the literature. Resuscitation.

[24] Berdowski J (2009). Importance of the first link: description and recognition of an out-of-hospital cardiac arrest in an emergency call. Circulation.

[25] Dami F (2010). Introducing systematic dispatcher-assisted cardiopulmonary resuscitation (telephone-CPR) in a non-Advanced Medical Priority Dispatch System (AMPDS): Implementation process and costs. Resuscitation.

[26] Kuisma M (2005). Emergency call processing and survival from out-of-hospital ventricular fibrillation. Resuscitation.

[27] Lewis M, Stubbs BA, Eisenberg MS (2013). Dispatcher-assisted cardiopulmonary resuscitation: Time to identify cardiac arrest and deliver chest compression instructions. Circulation.

[28] Roppolo LP (2009). Dispatcher assessments for agonal breathing improve detection of cardiac arrest. Resuscitation.

[29] Vaillancourt C (2015). Cardiac arrest diagnostic accuracy of 9‑1-1 dispatchers: A prospective multi-center study. Resuscitation.

[30] Ma MH (2007). Evaluation of emergency medical dispatch in out-of-hospital cardiac arrest in Taipei. Resuscitation.

[31] White L (2010). Dispatcher-assisted cardiopulmonary resuscitation: Risks for patients not in cardiac arrest. Circulation.

[32] Hallstrom AP (2003). Dispatcher assisted CPR: Implementation and potential benefit. A 12-year study. Resuscitation.

[33] Oman G, Bury G (2016). Use of telephone CPR advice in Ireland: Uptake by callers and delays in the assessment process. Resuscitation.

[34] Van Vleet LM, Hubble MW (2012). Time to first compression using Medical Priority Dispatch System compression-first dispatcher-assisted cardiopulmonary resuscitation protocols. Prehosp Emerg Care.

[35] Hallstrom A (2000). Cardiopulmonary resuscitation by chest compression alone or with mouth-to-mouth ventilation. N Engl J Med.

[36] Heward A, Damiani M, Hartley-Sharpe C (2004). Does the use of the Advanced Medical Priority Dispatch System affect cardiac arrest detection?. Emerg Med J.

[37] Stipulante S (2014). Implementation of the ALERT algorithm, a new dispatcher-assisted telephone cardiopulmonary resuscitation protocol, in non-Advanced Medical Priority Dispatch System (AMPDS) Emergency Medical Services centres. Resuscitation.

[38] Besnier E (2015). Dispatcher-assisted cardiopulmonary resuscitation protocol improves diagnosis and resuscitation recommendations for out-of-hospital cardiac arrest. Emerg Med Australas.

[39] Bohm K (2009). Tuition of emergency medical dispatchers in the recognition of agonal respiration increases the use of telephone assisted CPR. Resuscitation.

[40] Rea TD (2010). CPR with chest compression alone or with rescue breathing. N Engl J Med.

[41] Svensson L (2010). Compression-only CPR or standard CPR in out-of-hospital cardiac arrest. N Engl J Med.

[42] Huepfl M, Selig HF, Nagele P (2010). Chest-compression-only versus standard cardiopulmonary resuscitation: a meta-analysis. Lancet.

